# PYCR1 is Associated with Papillary Renal Cell Carcinoma Progression

**DOI:** 10.1515/med-2019-0066

**Published:** 2019-08-14

**Authors:** Qiu-Li Wang, Ling Liu

**Affiliations:** 1 Department of Nephrology, Jining NO.1 People’s Hospital, Jining, 272100, Shandong, China; 2 Department of Nephrology, Jining NO.1 People’s Hospital , No.6 Jiankang Road, Jining, 272100, Shandong, China

**Keywords:** Papillary renal cell carcinoma, PYCR1, Prognosis, Akt/mTOR

## Abstract

**Objective:**

We aimed to determine the function of pyrroline-5-carboxylate reductase 1 (PYCR1) on progression of papillary renal cell carcinoma (PRCC) and related mechanism.

**Methods:**

The TCGA database provided us expression profiles of PYCR1 and overall survival rates. Small interfering RNA (siRNA) was used to knockdown PYCR1; quantitative real-time polymerase chain reaction (qRT-PCR) and western blotting were conducted to identify the expression levels of mRNA and protein. The cell counting kit-8 (CCK-8) and colony formation assays were used to explore cell viability in Ketr-3 cells. The migration and invasion of Ketr-3 cells were investigated by transwell assays.

**Results:**

We found that PYCR1 was over-expressed in PRCC tissues and cells, causing poor outcomes. Moreover, reduction of PYCR1 played a negative role on cell proliferation, migration and invasion in tumor cells. The important Akt/mTOR pathway proteins, phosphorylated Akt (p-Akt) and phosphorylated mTOR (p-mTOR), also showed lower levels compared with control groups.

**Conclusion:**

These findings showed that disordered expression of PYCR1 could modulate PRCC progression through the Akt/mTOR pathway, implying a theoretical basis for PYCR1 as a potential therapeutic target in future clinical PRCC treatment.

## 1 Introduction

Renal cell carcinoma (RCC) is the most frequent cancer in the kidney, responsible for approximately 3% of all malignant cancer cases [1]. Complicated reasons make the treatment of RCC difficult, and multiple variants exacerbate this dilemma [2]. As the second most common cancer of kidney, papillary renal cell carcinoma (PRCC) accounts for about 20% of RCC [1, 3]. There are two subtypes of PRCC: papillary type 1 and papillary type 2 [4]. Linehan *et al*. have established that type 1 is associated with the methylethyltryptamine (MET) gene, while type 2 is associated with cyclin-dependent kinase Inhibitor 2A (CDKN2A), SET domain containing 2 (SETD2), and transcription factor binding to IGHM enhancer (TFE3) genes [5]. Considering the above relationships, it is essential to examine efficient gene targets for PRCC.

PYCR1 plays an important role in proline biosynthesis and conversion of pyrroline-5-carboxylate (P5C) into proline also depends on the involvement of PYCR1 [6]. Proline is a unique amino acid with a secondary amine and is non-essential in humans, which has been recognized as a structural disruptor and indicator of various pathological stresses during tumorigenesis [7, 8]. Proline could be generated by pyrroline-5-carboxylate reductase (PYCR) or ornithine cyclodeaminase [9]. Moreover, previous studies indicate that proline is a tumor inhibitor for cell viability, apoptosis in kidney cancer and oral cancer, via regulating reactive oxygen species (ROS) [10, 11, 12, 13]. Given this theoretical basis, we have reason to believe that PYCR is essential for tumorigenesis. There are three homologues including PYCR1, PYCR2 and PYCR3. Feng *et al*. confirm a close relationship between PYCR1 expression and non-small-cell lung carcinoma (NSCLC) progression [14]. Additionally, similar functions are verified in other tumors, such as breast cancer [15], lung cancer [14, 16], and prostate cancer [6]. However, the role of PYCR1 in PRCC is still unclear, although targeted drug treatment is regarded as an effective method for delaying cancer metastasis [17, 18].

In the present investigation, we first aimed to characterize the function of PYCR1 in PRCC. The expression level of PYCR1 in tumor tissues and cells was assessed, and prognosis relying on its expression level also was analyzed using statistical methods. Cell behaviors were measured using vitro experiments and the underlying mechanism was explored by key protein detection. Taken together, these data may provide new mechanistic insights into the function of PYCR1 for PRCC treatment in future.

## 2 Materials and methods

### 2.1 Cell lines and cell culture

The American Type Culture Collection (Manassas, VA, USA) provided us with cell lines including HRCE, 786-O, OS-RC-2, Ketr-3 and A498. RPMI-1640 medium (Thermo Fisher Scientific, Inc., Waltham, MA, USA) was used to culture these cell lines and environmental conditions were set at 37˚C and 5% CO_2_. Additionally, 10% fetal bovine serum (FBS) (Thermo Fisher Scientific, Waltham, MA, USA), 100 U/mL penicillin and 100 μg/mL streptomycin (Thermo Fisher Scientific, Waltham, MA, USA) were added to RPMI-1640 medium for cell growth.

### 2.2 Real-time PCR analysis

To determine the expression of PYCR1 in all cell lines, we isolated total RNA using Trizol (Thermo Fisher Scientific, Inc., Waltham, MA, USA). Then SuperScript III RNase H Reverse Transcriptase (Thermo Fisher Scientific, Inc., Waltham, MA, USA) was utilized to reverse transcribe total RNA into cDNA. PCR reactions were carried out on a Bio-Rad Connect Real-Time PCR machine with SYBR Green PCR Master Mix (Thermo Fisher Scientific, Inc., Waltham, MA, USA). The PCR procedure was run as follows: 95˚C for 5min, 95˚C for 30s with 40 cycles, 60˚C for 45s and 72˚C for 30min. The primer sequences: PYCR1: F, 5’-TGACCAA-CACTCCAGTCGTG-3’, R, 5’-GTCCAGCTTCACCTTGTCCA-3’; GAPDH: F, 5’-GGAGCGAGATCCCTCCAAAAT-3’, R, 5’-GGCT-GTTGTCATACTTCTCATGG-3’. The relative expression of PYCR1 was assessed by 2^-ΔΔCt^ analysis and GAPDH was regarded as standard control.

### 2.3 Cell transfection

Lipofectamine2000 transfection Kit (Thermo Fisher Scientific, Inc., Waltham, MA, USA) was used to inject si-PYCR1 into cells according to the manufacturer’s instructions. In order to control the unique variables and nonspecific interference, si-con without target was used as internal control. The sequences were as follows: PYCR1 siRNA 1 : F: 5’-CTTCATCCTGGATGAAAT-3’, R: 5’-GAAG-TAGGACCTACTTTA-3’; siRNA 2 : F: 5’-TGCTCATCAACGCT-GTGG-3’, R: 5’-ACGAGTAGTTGCGACACC-3’; si-con: F: 5’-AATTCTCCGAACGTGTCACGT -3’, R: 5’-TTAAGAGGCTTG-CACAGTGCA-3’. Primers were synthesized by GenePharma Co., Ltd (Shanghai, China).

### 2.4 Cell counting kit-8 assay

CCK-8 kit (Dojindo Molecular Technologies, Inc., Kumamoto, Japan) was used to investigate cell vitality. Transfected cells were placed into 96-well plates with 1,000 cells per well and incubated in a carbon dioxide incubator. 10 μL CCK-8 regent was added before cell activity was examined at 0 h, 24 h, 48 h and 72 h. Subsequently, cells were cultured at 37˚C for 1.5 h and then the OD values were read under a microscope at 450 nm.

### 2.5 Colony formation assay

Colony formation assays were used when the cells entered the log phase and these cells were trypsinized to make into suspensions. Cell suspensions were put into 60 mm dishs which contained 5 mL warmed medium at a density of 1,000 cells/dish and maintained for two weeks until clones appeared on the culture dish at 37˚C with 5% CO_2_. After washing with 1×PBS, cells were fixed and stained with 4% paraformaldehyde and 0.1% crystal violet respectively. Finally, the clones were counted by naked eye.

### 2.6 Transwell assays

Transwell assays were performed to explore cell migration and invasion. For cell invasion, Matrigel was diluted with medium six times after overnight thawing and then put into the upper chamber. Cells (1×105/well) were seeded into the upper chamber pre-coated with Matrigel and to the bottom chamber 500 μL complete medium was added . After 24 h incubation, cells that did not invade were removed by cotton swabs while invasive cells were fixed using 4% paraformaldehyde for 30 min, stained by 0.1% crystal violet for 20min. Next, PBS was used to wash off the floating color and the status was imaged under an optical microscope. Cells were calculated in several random fields. For cell migration, the density was 5, 000 cells per well and the chamber without Matrigel was utilized. The experiment was repeated at least three times and all the results were present as mean ± standard deviation (SD).

### 2.7 Western blotting

Proteins were extracted from transfected cells with RIPA buffer and quantified using a BCA kit (Beyotime, Shanghai, China). Each tank was filled with 20 μg proteins and then 12% SDS-PAGE was used to separate proteins. When proteins were transferred onto PVDF membrane, 5% non-fat milk was utilized to block these membranes for 1 h. Subsequently, PVDF membranes were incubated with primary antibodies (1:1,000; Cell Signaling Technology, Danvers, MA, USA) against Akt, p-Akt, mTOR, p-mTOR and GAPDH overnight at 4˚C. After washing with 1×TBST three times, PVDF membranes were cultured with secondary antibodies (1:5,000; Cell Signaling Technology, Danvers, MA, USA). Protein images were captured through ECL and the relative expression of protein bands were measured by Quantity One software.

### 2.8 Statistical analysis

Data analyses were performed with SPSS22.0 and Graph-Pad Prism version 5.0. Student’s t-test was conducted to compare the difference in two samples and one-way ANOVA with Dunnett’s post hoc test utilizing in diverse groups. Pearson’s analyses were applied to reveal the association between PYCR1 and PYCR2 or PYCR3. Significant statistical differences were defined as P < 0.05. All these results were expressed as the mean ± standard deviation (SD) in at least three replications. Ethical approval: The conducted research is not related to either human or animal use.

## 3 Results

### 3.1 PYCR1 is elevated in papillary renal cell carcinoma and correlates with poor outcome

**Figure 1:**
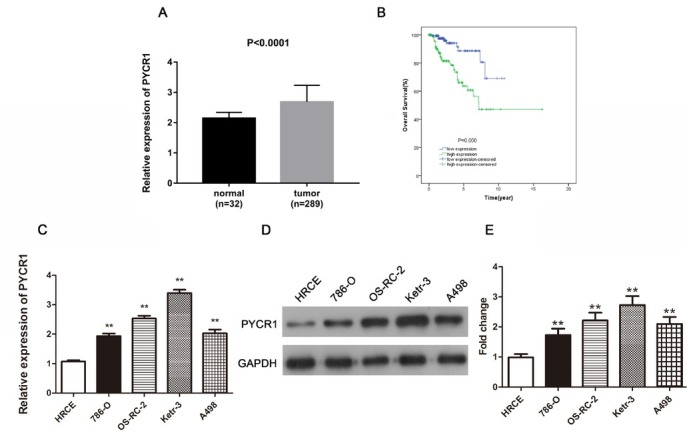
PYCR1 is promoted in PRCC tissues and cells, meanwhile up-regulated PYCR1 is related to poor prognosis in PRCC patients. (A) The relative expression of PYCR1 in PRCC tissues compared to normal tissues. (B) The overall survival curve in PRCC patients with high and low expression of PYCR1. (C) and (D) Comparison of PYCR1 expression in diverse cell lines through qRT-PCR and western blot analyses. (E) The quantification of protein expression level. All the data are expressed as mean ± SD and each test was performed in three times. (**P < 0.01). Supplement Figure 1: The correlation between PYCR2/PYCR3 expression and PYCR1 expression or survival rates of PRCC patients. (A) PYCR2 was over expressed in PRCC tissues consisting of 32 normal cases and 289 PRCC cases. (B) Survival curve showed the connection between PYCR2 expression and PRCC. (C) PYCR3 was increased in PRCC tissues including 32 normal cases and 289 PRCC cases. (D) The survival curves analysis on the basis of PYCR3 expression. (E) and (F) PYCR1 expression was closely linked with PYCR2/PYCR3 by the Pearson’s analyses.

To determine PYCR1 expression level and confirm association between PYCR1 expression level and papillary renal cell carcinoma, we first analyzed the expression level of PYCR1 in PRCC tissues and corresponding tumor cell lines. Data about PYCR1 expression in PRCC tissue was downloaded from TCGA database and revealed that compared to normal tissues, PYCR1 showed a higher level in the respect of mRNA (Fig.1A, P < 0.0001). The qRT-PCR and western blotting analyses also demonstrated PYCR1 expression was improved in different degrees in various cell lines of PRCC in comparison with human normal cell line HRCE, among which Ketr-3 cell line was most enhanced (Fig.1C-E, P < 0.01). Thus, the Ketr-3 cell line with highest PYCR1 expression was selected in the subsequent experiments. Due to the fact that PYCR1 was up-regulated in PRCC tissues and cells, we evaluated the relationship of PYCR1 and tumor prognosis in PRCC. The patients with PRCC with low PYCR1 expression showed a better outcome than those patients with high PYCR1 expression (Fig.1B, P < 0.001). The supplemented Fig.1 validated the relationship between PYCR homologous (PYCR1, PYCR2 and PYCR3) and PRCC. Taken together, these results suggested PYCR1 may exist as a carcinogenic factor in PRCC.

### 3.2 Reduction of PYCR1 hinders cell proliferation and colony formation of Ketr-3 cells

**Figure 2:**
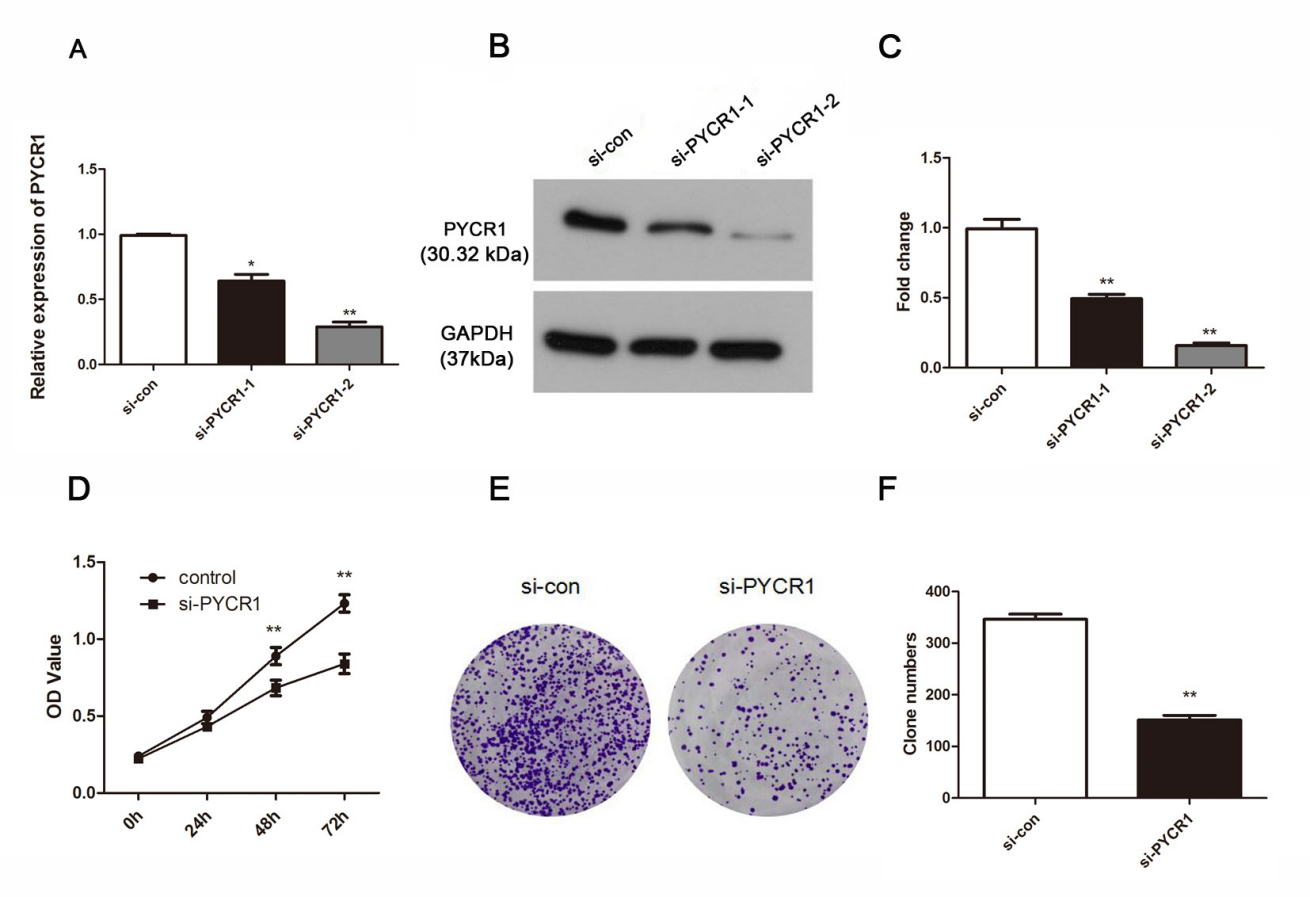
SiRNA strategy suppresses the PYCR1 expression successfully and retards cell viability in Ketr-3 cells. (A and B) The effective rates were detected by qRT-PCR analysis and western blot. (C) The fold change of western blot results. (D) Cell proliferation was measured using CCK-8 assay. (E) Colony formation was measured and (F) the number of clone was counted. The data are expressed as mean ± SD in triplicates. (*P < 0.05, **P < 0.01)

In order to explain the role of PYCR1 accurately, we knocked down PYCR1 using siRNA in Ketr-3 cells. The effective reduction was exhibited at both the mRNA level and protein level, especially si-PYCR1-2 (Fig.2A-C, P < 0.05). According to these results, CCK-8 and colony formation assays were performed subsequently to detect the role of PYCR1 on cell vitality. As shown in Fig.2D, knockdown of PYCR1 blocked cell growth remarkably 48 h and 72 h after transfection (P < 0.01). Furthermore, the colony formation activity of Ketr-3 cells treated with si-PYCR1-2 was also examined. This investigation indicated that low-expression of PYCR1 weakened cell colony formation and the clone numbers verified this result (Fig.2E and 2F, P < 0.01).

### 3.3 Si-PYCR1 plays a suppressive role on cell migration and invasion in Ketr-3 cells

**Figure 3:**
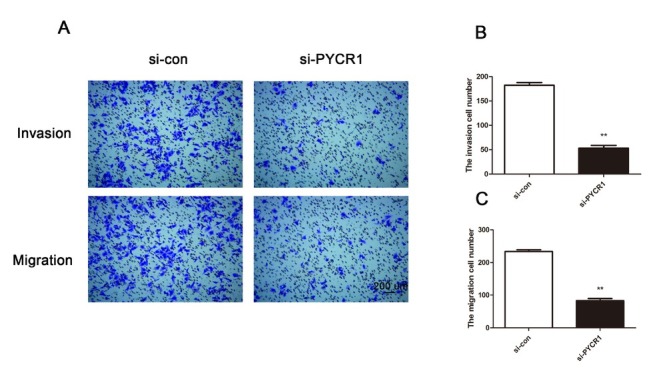
PYCR1 knockdown inhibits cell migration and invasion in Ketr-3 cells. (A) Transwell assays showed the difference of cell migration and invasion between si-con group and si-PYCR1 group. (B) and (C) The migratory and invasive cells were calculated. Bar = 200 μm. Every experiment was conducted three times. (**P < 0.01)

Next, transwell assays were implemented to explore cell migration and invasion in Ketr-3 cells after PYCR1 knockdown. The experimental results demonstrated that deficiency of PYCR1 inhibited Ketr-3 cell activity in the aspect of invasion while there was no significant difference in the control group (Fig.3A). Meanwhile, compared with the si-con group, the mean number of invasive cells in the experimental group was also significantly decreased (Fig.3B, P < 0.01). Migratory activity exhibited the similar tendency as invasion (Fig.3A and 3C, P < 0.01). These findings identify that si-PYCR1 might have a negative effect on migration and invasion in Ketr-3 tumor cells.

### 3.4 Akt/mTOR pathway is involved in the effect of PYCR1 knockdown on PRCC cell functions

**Figure 4:**
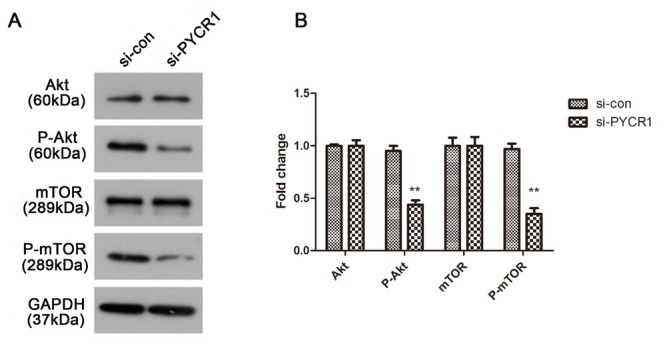
Silenced PYCR1 has a negative role on Akt/mTOR pathway in PRCC. (A) The expression level of Akt, p-Akt, mTOR and p-mTOR, with GAPDH considered as control. (B) Relative protein expression was quantified and depicted in column diagram. All the tests were carried out in triplicates independently. (**P < 0.01)

Considering the function of PYCR1 knockdown on PRCC cells, we further studied the underlying molecular mechanism involved in PRCC. We found that the introduction of si-PYCR1 dramatically hindered p-Akt and p-mTOR protein expression in Ketr-3 cells when compared to the si-con group, but that Akt and mTOR implied no difference (Fig.4A). Fig.4B describes the quantitative results of protein expression (P < 0.01). All the data pointed to PYCR1 manipulating the Akt/mTOR pathway to affect cell proliferation, migration and invasion in PRCC.

## 4 Discussion

We discovered that PYCR1 was over-expressed in human PRCC tissues compared with controls. Highly regulated PYCR1 expression led to poor prognosis in PRCC patients. All the data suggest that PYCR1 could promote the tumorigenesis of PRCC in the view of cell proliferation, migration and invasion.

Previously, metabolism is required in tumors and mediates cellular processes, such as proliferation, redox homeostasis and reprogramming [19]. Recent studies have shown that proline metabolism plays an important function in humans, especially in cancer [20], suggesting that disruption of proline metabolism may be an attractive strategy for tumor treatment. Intensive research illustrates that PYCR1 was highlighted in the biosynthesis of proline in various tumors. For instance, PYCR1 could cooperate with isocitrate dehydrogenase 1 (IDH1) to promote cancer cell survival in glioma and esophageal squamous cell cancer [21, 22]. In 2014, Nilsson *et al*. searched for different genes that were consistent in nineteen cancers and found that PYCR1 is proposed as a novel enzyme [23]. In addition to its involvement in cancer, PYCR1 could also be involved in other disease related processes. Oxidative stress was associated with PYCR1 as well as PYCR2 [24, 25]. Reduction of PYCR1 activity could result in cell apoptosis and damage to mitochondria [26]. Moreover, the homolog of PYCR1, PYCR2, induces mutations related to microcephaly and hypomyelination [27, 28].

We performed experiments *in vitro* and found that PYCR1 could accelerate the progression of PRCC. There is a study about skin cancer that identified that PYCR2 was abundantly present in melanoma cells and regulated the Akt/mTOR pathway [29]. Epidermal Growth Factor Receptor (EGFR) inhibitor and VEGF/mTOR inhibitors were all applied to treat RCC which contains PRCC [30, 31].

In order to determine how PYCR1 regulates the physiological processes of tumor cells, we first examined the effect of silencing PYCR1 on the Akt/mTOR pathway. The results of western blotting showed p-Akt and p-mTOR were impaired owing to knockdown PYCR1. On the contrary, the normal control exhibited no difference. However, here we did a preliminary investigation about the relationship between PYCR1 and PRCC. To thoroughly understand the underlying mechanism, a series of in-depth studies including in vivo experiments need to be followed up.

In total, all the data in this present study confirm that PYCR1 was highly regulated in PRCC and its over-expression could induce poor prognosis in patients with PRCC. Based on the results of the silencing tests, PYCR1 stimulated cell behaviors including proliferation, migration and invasion. The vitality of colony formation was also inhibited because of silencing PYCR1. The key proteins, phosphorylated Akt and mTOR, revealed a downtrend at the level of protein expression level. We predict that PYCR1 might be an independent risk element for patients prognosis in PRCC via regulating Akt/mTOR pathway.
